# Prolonged nightly fasting in older adults with memory decline: A single-group pilot study exploring changes in cognitive function and cardiometabolic risk factors

**DOI:** 10.1017/cts.2024.676

**Published:** 2024-12-12

**Authors:** Dara L. James, Linda K. Larkey, Molly Maxfield, SeungYong Han, Edward Ofori, Alex E. Mohr, Nanako A. Hawley, Kate Alperin, Erica Ahlich, David E. Vance, Dorothy D. Sears

**Affiliations:** 1 Edson College of Nursing and Health Innovation, Arizona State University, Phoenix, AZ, USA; 2 Kaiser Permente, California, LA, USA; 3 College of Health Solutions, Arizona State University, Phoenix, AZ, USA; 4 Department of Psychology, University of South Alabama, Mobile, AL, USA; 5 College of Liberal Arts and Sciences, Arizona State University, Tempe, AZ, USA; 6 School of Nursing, University of Alabama Birmingham, Birmingham, AL, USA

**Keywords:** Intermittent fasting, dementia, cognition, circadian rhythm, aging

## Abstract

**Introduction:**

Older age significantly increases risk for cognitive decline. A growing number of older adults (≥ 65 years) experience cognitive decline that compromises immediate and/or long-term health. Interventions to mitigate cognitive decline are greatly needed. Intermittent fasting aligned with innate circadian rhythms is associated with health benefits and improved circadian rhythms; here, we explore impacts on cognition and cardiometabolic outcomes.

**Methods:**

We conducted a single-group, pre-/post-pilot study to explore an 8-week prolonged nightly fasting intervention (14 h fasting/night) among adults 65+ years with self-reported memory decline. We explored changes in cognitive function, insomnia, and cardiometabolic risk factors. Intervention engagement/adherence were assessed. The intervention was delivered fully remotely; participants completed their fasting protocol at home and were not required to come into the lab.

**Results:**

In total, 20 individuals signed consent and 18 participants completed the study. Participants were mean age 69.7 years, non-Hispanic White (89%), predominantly female (95%), married (50%), and employed (65%). Paired *t*-tests indicated an increase in cognitive function (Memory and Attention Phone Screener) (*p* = 0.02) with a medium effect size (Cohen’s *d* = 0.58) and a decrease in insomnia (Insomnia Severity Index) (*p* = 0.04) with a medium effect size (Cohen’s *d* = 0.52). Changes in BMI or diet quality were not observed. Engagement (66%–77%) and adherence (70%–100%) were high.

**Conclusion:**

These pilot findings suggest that prolonged nightly fasting, targeted to align food intake with circadian rhythms, may improve cognitive function and sleep among older adults. Fully powered, randomized controlled trials to test the efficacy of this non-pharmacological, low cost-to-burden ratio intervention are needed.

## Introduction

The USA median age is rising with greater than 55 million individuals being classified as older adults, defined as 65 years of age or older [1]. Normal aging includes notable changes and declines across all domains of cognitive function [2]. Age is the primary risk factor for cognitive decline and neurodegenerative disease, including mild cognitive impairment (MCI), Alzheimer’s disease (AD), and Alzheimer’s disease-related dementias (ADRD); thus, cognitive decline/disease is a significant public health concern [3]. The total number of people diagnosed with neurodegenerative diseases has risen steeply, with recent statistics estimating that greater than 12 million Americans will be diagnosed with dementia by the year 2050 [4]. AD is the most prevalent form of dementia and is a progressive, neurodegenerative disease characterized by impaired memory, judgment, and the ability to carry out activities of daily living [5]. Currently, there are no cures for AD, the progressive nature of which, over time, often requires the need for a caregiver [6]. Given the growing older adult population and multifaceted impact (e.g., emotional, physical, and financial) of cognitive decline and neurodegenerative diseases, it is critical to understand the landscape of changes across cognitive function (multi-domain and global) associated with age in an effort to support reduced disease risk and to maintain functional independence and quality of life.

Characteristically, age-related cognitive decline is identified as the progressive and gradual accumulation of detrimental changes in both the brain structure and brain function over time [3]. Age-related cognitive decline impacts multi-domain and global cognitive function, including domains of episodic memory, language skills (verbal fluency, name and word comprehension), visuospatial abilities, and executive functions [2]. A diagnosis of dementia later in life is likely driven by a conglomerate of both non-modifiable (e.g., age and genetics) and modifiable (e.g., diet, exercise, smoking status, and alcohol consumption) lifestyle factors, significantly impacting disease incident and possible progression. The current evidence-based modifiable lifestyle factors that have demonstrated modulation and improvement of cognitive function and disease trajectory include diet, exercise, and cognitive stimulation/engagement [7].

Accumulating evidence demonstrates the significant impact of healthy lifestyle behaviors as compelling strategies to prevent and attenuate age-related cognitive decline and further, potentially delay the onset of, or attenuate the progression of, clinical diagnoses including MCI and AD/ADRD [8]. Repeatedly, research promotes the initiative and ongoing efforts targeting lifestyle interventions to modify and postpone the trajectory of age-related cognitive decline prior to the potential onset of a clinical and irreversible diagnosis [3,9]. Given that lifestyle behaviors such as poor diet, lack of exercise, and poor/reduced sleep are known risk factors for progressing beyond MCI, these have been suggested as potential independent and interdependent targeted therapeutic approaches. However, to date, efficacious therapies and programs based on lifestyle are limited and often suffer from feasibility constraints. A systematic review and meta-analysis examining exercise as an intervention for improving cognition in AD patients was inconclusive as 25% of studies did not see an improvement in cognitive functioning [10]. A systematic review revealed that most current treatments targeting sleep disturbance in AD are ineffective [11].

As an alternative dietary strategy, intermittent fasting includes specified periods of hours and/or days with of little or no caloric consumption [12,13]. Traditionally, intermittent fasting does not include caloric restriction as the targeted focus is on timing of caloric consumption; however, intermittent fasting protocols may include a secondary emphasis on calories or macronutrients [12,14]. Recently, the use of intermittent fasting in studies of animals and humans has been investigated to better understand the appropriate application, health/disease-related outcomes and mechanisms of change [13]. For purposes of the current work, and specific to circadian rhythms, we further explore the use of prolonged nightly fasting. The practice of prolonged nightly fasting is considered a subset of time-restricted eating aligned with circadian rhythms such that calories (i.e., food and drink) are consumed during the daytime hours, and fasting occurs during the night [15]. Metabolically and biologically, the human body is designed to eat during the daytime (i.e., with light) hours and be at rest during the nighttime (i.e., without light) hours [16].

Circadian rhythm misalignment is both associated with and a risk factor for AD [17], in part due to the effects on sleep, inflammation, and metabolic function [18]. Circadian rhythms activate and deactivate different brain activities during the sleep/wake cycles; however, when sleep is disrupted due to circadian rhythm misalignment, or desynchronization, brain activities are inappropriately activated, and can lead to impaired cognition and memory [19]. Specifically, circadian rhythm misalignment may negatively impact inhibitory control, working memory, and task switching [20]. Additionally, during sleep, blood pressure typically drops, and the body relaxes; if the circadian rhythm is disrupted, blood pressure cannot regulate appropriately and may lead to inflammation. Chronic inflammation is linked to cognitive decline and, over time, may contribute to the development or progression of neurodegenerative diseases.

Insomnia, a common sleep disorder, is characterized by issues with falling or staying asleep and is associated with decreased functioning during wakefulness: fatigue, mood disruption, increased risk for developing mood disorders (i.e., depression and anxiety), and impaired cognitive performance [21]. Insomnia is more prevalent and severe among older adults as compared to young adults [22] and, further, has been found to impair attention, executive functioning, and slower reaction time compared with older adults without insomnia [21]. Older adults with chronic insomnia performed significantly poorer on tasks of memory span, attention, time estimation, executive functioning, processing speed, and visuospatial skills [23]. Insomnia can have detrimental effects on cognitive function and is associated with increased incidence and pathogenesis of AD [24].

Aimed at aligning eating times with the circadian rhythm, prolonged nightly fasting has demonstrated benefits including improvement in glucose metabolism [25] and sleep [26], both of which are factors related to cognitive health, function, and disease. Furthermore, prolonged nightly fasting may support improvements in cognitive health through mechanisms of autophagy (i.e., cells recycling damaged part of itself) and the initiation of protective responses to buffer oxidative stress and inflammation [14,27] While recent investigations have explored prolonged nightly fasting in the context of physical functioning [28] and nutritional status [29] in older individuals, to the best of our knowledge, none have assessed cognitive measures.

The purpose of this single-group, pre-/post-pilot study titled “Think FAST,” was to explore the use of a 14-h, 8-week prolonged nightly fasting protocol in a group of older adults (≥ 65 years old) with self-reported memory decline on the primary outcome of cognitive function, and secondary outcomes of cardiometabolic risk factor (i.e., BMI) and both sleep and diet quality. In the context of a pilot study (i.e., not hypothesis driven), we anticipated that prolonged nightly fasting would improve cognitive function and select related cardiometabolic outcomes (i.e., BMI) and both sleep and diet quality among a group of older adults with self-reported memory decline. Further, we aimed to understand participant adherence with the prolonged nightly fasting protocol.

## Materials and methods

### Study overview

The current study was conducted as a remotely delivered, single-group, 8-week intervention pre-/post-pilot to explore changes in cognitive function and cardiometabolic risk factors. Older adults ( ≥65 years; *N* = 18) with self-reported memory decline engaged in 8 weeks, remotely delivered, prolonged nightly fasting. The primary outcome, cognitive function, was assessed via composite score of the Memory and Attention Phone Screener (MAPS). Factors known to be associated with cognitive function were also assessed at pre- and post-intervention, including sleep and diet quality. This study was approved by the Institutional Review Board at affiliated university and registered on Clinicaltrials.gov (NCT04938778). Enrolled participants provided written consent.

### Eligibility and recruitment

Participant eligibility was based on the following inclusion and exclusion criteria as listed below. Inclusion criteria included: 1) self-reported “memory not as good as it used to be;” 2) ≥65 years old; 3) lived in the city of the funding university; 4) able to speak/understand English; 5) owned a scale for at-home body weight measurements; and 6) owned a smartphone and had access to Zoom and WiFi. Exclusion criteria included: 1) previously diagnosed with AD or another type of dementia, psychological, psychiatric, or neurological diseases; 2) had been routinely fasting for 14+ h a night; 3) had been diagnosed with a clinical eating disorder in the previous 20 years; 4) had a clinical diagnosis of diabetes and/or any other medically-based reason that would preclude them from safely participating in fasting; 5) worked a night shift job; or 6) responded “no” to the eligibility question: “Do you feel your memory is becoming worse?”

Twenty-one participants were recruited from July 2021 through March 2022. Participants were recruited through the following strategies based in the area of the funding university: university newsletters/announcements, Facebook, fliers placed in the community and at local farmer’s markets. Prospective participants initially contacted the study team via email or study specific phone number. Eligibility screening occurred via brief phone call (∼5 min) with trained study staff and was scheduled at participants’ day/time preference. Individuals who were eligible and interested in joining the study were emailed a consent form to sign and return via email, prior to enrollment.

### Procedures

All data (pre- and post-intervention) were collected remotely via Zoom with participants and trained study staff (one-on-one meetings). During an online Zoom meeting, data collected for the self-reported measures were entered directly into REDCap electronic data capture tools [30] by study staff. Participants were asked the questions on the self-reported measures during their baseline and post-intervention data collection appointments. Participants were also asked to self-report their last recorded height and weight to calculate BMI at baseline and post-intervention. Study staff administered the MAPS via Zoom pre- and post-intervention; responses were recorded on paper (by study staff) and saved in deidentified participant folders (data were later entered into excel for analysis). The MAPS was designed for the present study and includes items assessing verbal memory (immediate and delayed member for a word list), attention (reverse digit span), processing speed (Oral Trail Making Test A), set-shifting (Oral Trail Making Test B), working memory (serial subtraction from 100), verbal fluency (category and semantic), and orientation. The MAPS includes items from other cognitive screens (the Colorado Cognitive Assessment [31] and the Telephone Montreal Cognitive Assessment [32]) and tests (the Oral Trail Making Test [33]), which were specifically chosen to allow administration over Zoom. Items were also selected to provide a greater range of scores compared to a phone-administered screen, like the Telephone Montreal Cognitive Assessment, which is designed to detect objective neurocognitive impairment, and we did not anticipate high rates of mild cognitive impairment or dementia among participants.

### Prolonged nightly fasting intervention

Participants were asked to engage in an 8-week prolonged nightly fasting intervention of 14 hper night for 6 days each week. One “day off” was allowed per week based on participant preference; day of week could vary week to week as selected by the participant. Participants were asked to begin their 14-h prolonged nightly fast starting no later than 8:00 pm, followed by a 10-h eating window during the day time. During the fast, participants were permitted to drink water, coffee, or tea (without milk products or sweeteners/artificial sweeteners). Participants were free to choose the 10-h eating window that worked best for them, conditional to their fast starting no later than 8:00 pm. Participants were asked to consume foods and beverages as they normally would during their eating window (i.e., no changes to diet quantity or quality).

Participant information packets were printed and mailed to each participant prior to the start of their intervention period (prior to baseline data collection). Packets included a weekly and monthly paper calendar to track fasting dates and start/stop times. The fasting start/stop times for each of the 6 days per week were recorded by participants on these paper tracking sheets. Fasting dates and start/stop time information was collected by the study staff member each week during the participant/study staff weekly check-in phone calls (tracking sheets and information packets were not returned to the study staff).

### Measures

Validated self-report measures were used to assess variables. Additionally, due to the restrictions of the COVID-19 pandemic (i.e., no in-person data collection), we assessed cognitive function with the MAPS developed by a co-author and geropsychologist. The MAPS yields a composite score derived from previously validated cognition scales; additional information detailed below. All measures were collected at baseline and the 8-week intervention end point.

#### Demographics

Participant age, race, ethnicity, gender, marital status, employment, education, and income information were collected at baseline.

#### Memory Attention Phone Screener (MAPS)

The primary outcome of cognitive function was measured using the composite score of the MAPS. The MAPS was recorded via Zoom video conferencing technology for validation of responses, and responses were then transferred onto hardcopies and followed up with direct entry into REDCap. The MAPS was based on previously validated cognitive screens and adapted for the present study to be easily administered over Zoom during the COVID-19 pandemic. MAPS included brief measures of memory both immediate and delayed recall of a word list (3 trials with 7 words), working memory (Reverse Digit Span and serial 7s and 4 trials), processing speed (oral version Trail Making Test, counting to 25 as quickly as possible without missing numbers) [34], verbal fluency (semantic and phonemic), and basic orientation (date, day of the week, city and state currently in).

The MAPS scale was constructed by summing nine items measuring immediate (0 to 21) and delayed memory (0 to 7), orientation (0 to 6), digit span reverse (0 to 4) and working memory (0 to 3), oral trails A and B, and phonemic and semantic fluency. Two items for oral trails A [34] and B were reverse coded, with high scores indicating better cognitive performance. Additionally, we anticipated the 8-week prolonged nightly fasting intervention would be most impactful on working memory and set-shifting, which benefit from improved sleep quality [35]; we therefore included the oral version of the Trail Making Test to include assessment of attention set-shifting.

#### Insomnia Severity Index (ISI)

Insomnia symptoms and severity were measured using the Insomnia Severity Index (ISI) [36]. The 7-item scale is a measure for current sleep quality within the past 2 weeks. A 5-point Likert type scale (e.g., 0 = no symptom; 4 = severe symptom) was used to score each item; total score ranging from 0 to 28. Higher scores indicate clinically significant insomnia: 0–7 = no clinically significant insomnia, 8–14 = subthreshold insomnia, 15–21 = clinical insomnia (moderate severity), and 22–28 = clinical insomnia (severe) (Cronbach’s *α* = 0.84).

#### Rapid Eating Assessment for Participants Scale (REAP-S)

Diet quality was assessed using the with the Rapid Eating Assessment of Participants Scale (REAP-S) [37]. REAP-S contains 16 items, with the first 13 items assessing diet quality by measuring intake of foods such as whole grains, fruits, vegetables, fat, sugar, and foods containing calcium. REAP-S also includes three supplemental questions on ability to shop and cook as well as participant’s willingness to change their current diet. Each question is rated on a 1–3 scale (e.g., 1 = usually consumed, 2 = sometimes consumed, 3 = rarely/never consumed). The sum of the first 13 items yields a total score ranging from 13-39 with higher scores indicating higher diet quality (Cronbach’s *α* = 0.86).

#### Creature of Habit Scale (COHS)

Tendency toward routine was measured using the Creature of Habit Scale (COHS) “Routine” subscale consisting of 16 questions [38]. An example item is “I tend to like routine.” Items are scored on a 5-point Likert scale (e.g., 1 = strongly disagree, 5 = strongly agree). Scores are totaled and range from 16 to 80 with higher scores indicating more routine behaviors (Cronbach’s *α* = .89).

#### Self-report Body Mass Index (BMI)

Given the novel approach of this remotely delivered intervention initiated during the COVID-19 global pandemic, participant BMI was calculated by study staff using self-report height and weight provided via phone by participants at baseline and post-intervention.

#### Engagement and adherence

Engagement was measured by assessing the number of times over the course of the 8-week study that participants engaged in their once-weekly check-in with study staff (via email, phone, or text). Adherence was measured by calculating the number of reported days across the 8-week intervention (48 days total, one “cheat day” allowed per week) during which participants adhered to the 14-h nightly fast (for participants who reported a minimum of 75% of their fasting windows over the 8-weeks).

### Statistical analyses

Descriptive statistics are presented as means and standard deviations (see Table 1). Paired *t*-tests were used to explore whether differences in the means of each outcome measured at T1 and T2. Stata version 17.0 was used for data management and analysis.

**Table 1.** Participant demographics

Baseline characteristics (*N* = 20)	*n* (%)
Gender	
Female	19 (95)
Male	1 (5)
Latino, Chicana, or Mexican American	
Yes	1 (5)
No	19 (95)
History of MCI or AD/ADRD	
Yes	5 (25)
No	15 (75)
Marital status	
Married	10 (50)
Divorced, widowed, never married	10 (50)
Live independently without a caretaker/family member	
Yes	8 (42)
No	11 (58)
Employment status	
Full/part-time employed	12 (63)
Unemployed	7 (37)
Years of education	
<17 years	10 (50)
>17 years	10 (50)
Household income	
$35,000–$49,000	3 (15)
$50,000–$74,000	5 (25)
$75,000–$99,000	3 (15)
>$100,000	5 (25)
Declined to respond	4 (20)

*Note.* MCI = mild cognitive impairment; AD/ADRD = Alzheimer’s disease/Alzheimer’s disease-related dementias.

## Results

### Participants

**Figure 1.**
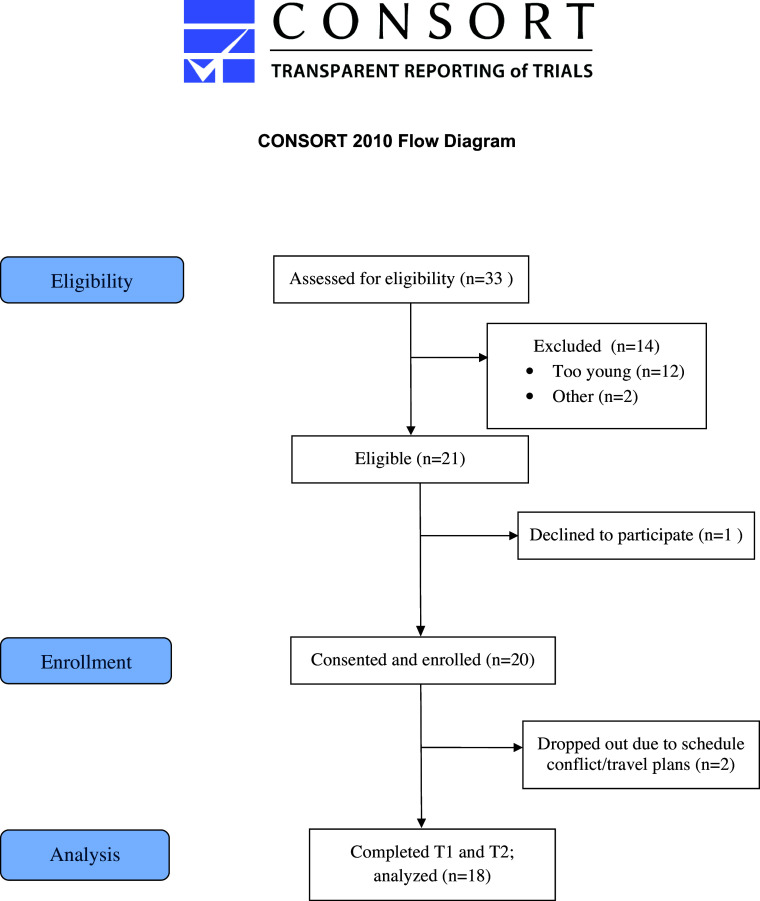
CONSORT flow diagram.

A total of 33 participants were screened for eligibility, 21 of whom were eligible. In total, 20 participants signed informed consent and enrolled in the study. Throughout the study duration, two participants dropped out of the study due to weekly schedule and/or travel conflicts (see Figure 1 for modified CONSORT flow diagram), yielding a total of 18 participants for pre-/post-intervention analysis. Study completion rate (i.e., the percentage of participants that completed the intervention from those that started the intervention) was 90%. Participant ages ranged from 61 to 84 years with mean age of 69.7 years old and had a baseline mean BMI of 29.5 kg/m^2^. The majority of study participants were non-Hispanic white (95%), predominantly female (95%), married (50%), employed (65%), and had an income ≥$50,000 (see Table 1 for baseline characteristics). The majority of participants (75%) did not have a history of MCI or AD/ADRD in their family. Primary and secondary study results are summarized in Table 2. Throughout the 8-week intervention, no adverse events or effects related to the intervention were reported.

**Table 2.** Primary and secondary outcomes at baseline (T1) vs. post-intervention (T2)

Outcome	T1 *M (SD)*	T2 *M (SD)*	*p*	*d*
BMI	29.51 (6.26)	28.27 (5.81)	0.200	0.31
Insomnia Severity Index (ISI)	6.72 (5.65)	5.00 (3.74)	0.043	0.52
ISI categories (n, %)				
Not clinically significant	10 (55.56)	13 (72.22)		
Subthreshold	7 (83.89)	5 (27.78)		
Clinical insomnia-moderate	1 (5.56)	0 (0)		
REAPS	21.61 (4.70)	21.89 (3.77)	0.747	0.08
Creature of Habit Scale	58.72 (10.95)	58.17 (10.75)	0.686	0.10
MAPS composite	30.47 (26.10)	42.35 (17.01)	0.02	0.58

BMI = body mass index; REAPS = Rapid Eating Assessment of Participants Scale; MAPS = Memory and Attention Phone Screener.

### Outcome measures

The primary outcome of cognitive function significantly improved after the 8-week prolonged nightly fasting intervention (*p* = 0.02) with a medium effect size (Cohen’s *d* = 0.58), as measured by the composite score by the MAPS. The 8-week prolonged nightly fasting intervention significantly decreased symptoms and severity of insomnia (*p* = 0.04) with a medium effect size (Cohen’s *d* = 0.52), as assessed by the Insomnia Severity Index (ISI). None of the other outcome measures were significantly changed after the intervention. A small effect size was calculated for BMI (Cohen’s *d* = 0.31), a negligible effect diet quality change (Cohen’s *d* = 0.08), and a negligible effect on changes in routine (Cohen’s *d* = 0.097).

### Engagement and adherence

Overall, both engagement with and adherence to the study protocol were strong over the course of the 8 weeks. Engagement rates for attending each of the 8 weekly check-in calls was 67%; engagement rates for attending 7 of the 8 weekly check-in calls was 78%. Adherence rates ranged from 70% to 100% for participants across the 8-week PNF intervention. Additionally, on average the majority of the study participants started their fasting window between 6:00 pm and 8:00 pm each of the six required nights per week.

## Discussion

Intermittent fasting may serve as a modifiable lifestyle intervention to improve domains of health specific to cognitive function and cardiometabolic risk. Our results support the implementation of an 8-week prolonged nightly fasting intervention to improve cognitive function and reduce insomnia severity/symptoms. Our pilot pre-/post-study did not find evidence of change in diet quality assessed by the REAPS, BMI, or habits/routine. A small effect size observed for change in BMI may indicate that a longer intervention duration could lead to BMI reduction. A 2023 study implementing a 16/8 (16 h of fasting followed by 8 h of an eating window) time-restricted eating protocol demonstrated improvements in cardiometabolic outcomes among female breast cancer survivors with respect to cardiovascular disease risk and metabolic syndrome status [39]. Improvements in cardiometabolic risk factors may require a longer fasting duration and/or a longer intervention period, and future work should explore protocol iterations to better understand how intermittent fasting may potentially improve cardiometabolic outcomes.

There is a critical need for non-pharmacological, behavioral lifestyle interventions to improve cognitive function, particularly among older adult populations, who are at a significantly increased risk for age-related cognitive decline and the potential diagnosis of neurodegenerative diseases including MCI and AD/ADRD. As highlighted in the current single-group pilot study, intermittent fasting, specifically, prolonged nightly fasting, may yield successful and promising results to improve cognitive function and, respectively, reduce insomnia severity and symptoms among older adults. This single-group pilot study provides preliminary evidence for the potential relationship between intermittent fasting, specifically, prolonged nightly fasting, and improved cognitive function and reduced symptoms/severity of insomnia among older adults with self-report memory decline.

In line with findings from the current pilot study, recent research highlights the potential for practices of intermittent fasting (prolonged nightly fasting; alternate-day fasting) to improve sleep behaviors related to insomnia (i.e., symptoms, severity) and sleep quality [15,40]. Our recent nationwide, remotely delivered randomized controlled trial demonstrated that compared to a health education control group, participants in the prolonged nightly fasting intervention group improved their sleep quality from pre- to post 8-week study [15]. Results from this pilot study support and extend the existing literature showing that intermittent fasting, in particular, prolonged nightly fasting, is associated with improved sleep outcomes. To date, most of this intermittent fasting research has been conducted in populations of individuals other than older adults [41]; here we expand this work with the targeted inclusion of a population of older adults (≤65 years). Moreover, while most of this research on intermittent fasting has focused on weight loss and metabolic-related outcomes [16,39,42,43], the novelty of the current work explores intermittent fasting in the context of the primary outcome of cognitive function and secondary outcome of insomnia. Findings from our recently completed work with a population of midlife adults living with stress and obesity demonstrated improvements in cognition from pre- to post-intervention using a prolonged nightly fasting regimen [15]. The current study adds to the existing body of literature with further exploration and understanding of intermittent fasting as a remotely delivered alternative dietary strategy to improve cognitive function.

### Strengths and limitations

A strength of this pilot study is the success in the remote delivery of the intervention. This study model led several others of our team members to successfully design and execute a nationwide, remotely delivered randomized controlled trial [15]. An additional strength was the successful completion on the pilot study during the COVID-19 pandemic, particularly with a population of individuals less likely to be enrolled in a clinical trial. There are several limitations of this pilot study. First, the sample size was small (*N* = 18) and included primarily females (95%) limiting the interpretation and generalizability of the findings. Additionally, our participant group lacked diversity with respect to gender, race, and ethnicity. Future work may address these limitations by utilizing additional and more diverse recruitment strategies. Notably, our single-group study lacked a control group for comparison in findings and/or demonstration of efficacy. The MAPS scale is in the process of being validated to further provide a valid and reliable remote delivery objective measure of cognitive function. In that regard, the use of the MAPS scale is a study strength as it is an objective measure of cognitive performance; as such, this provides a less biased estimate of actual cognitive changes rather than subjective (i.e., self-reported) cognitive function which can be biased or underestimated due to mood (i.e., depression), fatigue, and meta-cognitive deficits [44–46]. However, as subjective cognitive functioning (e.g., Cognitive Failures Questionnaire) reflects one’s perception of one’s cognitive abilities, it is often seen as a quality of life indicator as well in that if one perceives their cognitive abilities to be poor, this can be distressing [47]. Thus, future studies of prolonged nightly fasting should consider the use of subjective cognitive functioning as a secondary intervention outcome (e.g., Vance et al., 2022) [48].

## Conclusion

Results obtained from this current single-group pilot study suggest there are potentially important relationships between intermittent fasting, in particular, prolonged nightly fasting, and cognitive function, as well as insomnia symptoms and severity among older adult populations. The fact that sleep and BMI both improved and that these factors known to affect cognitive function provides initial support for the potential for intermittent fasting to operate across systems to leverage change. Future research will benefit from extending these results with a larger sample size and randomized controlled trial study design including a more diverse population. Additionally, future research may yield more significantly impactful findings with a longitudinal study design testing outcomes over multiple time points and examining the possible mediating roles of sleep and BMI. Reducing insomnia severity and symptoms through improved sleep management, in part driven by prolonged nightly fasting, may provide an easily deliverable and scalable way to improve cognitive function. Improved insomnia and cognitive function may help slow the progression of potential onset of neurodegenerative disease diagnosis. Innovative interventions that are feasible, low-cost/burden, and easy to disseminate are urgently needed to improve cognitive function and related cardiometabolic risk factors among older adults; prolonged nightly fasting, as a specified intermittent fasting regimen, offers promise for improved outcomes.
